# Supplementary material for: Exploration and Optimization of the Polymer-Modified NiOx Hole Transport Layer for Fabricating Inverted Perovskite Solar Cells
Source: Nanomaterials (Basel). 2024 Jun 19;14(12):1054. doi: 10.3390/nano14121054 (PMC11206296; doi:10.3390/nano14121054)
Supplement: Supplementary file 1 [file nanomaterials-14-01054-s001.zip › nanomaterials-3061647-supplementary.pdf]

# Supporting Information

# Exploration and optimization of the polymer-modified $NiO_x$ hole transport layer for fabricating inverted perovskite solar cells

You-Wei Wu, Ching-Ying Wang and Sheng-Hsiung Yang*

*Institute of Lighting and Energy Photonics, College of Photonics, National Yang Ming Chiao Tung University, No.301, Section 2, Gaofa 3rd Road, Guiren District, Tainan 711010, Taiwan R.O.C.*

*Correspondence: yangsh@nycu.edu.tw

## Experimental section

*1. Materials*

The FTO-coated glass substrates were bought from Ruilong Optoelectronics Technology Co., Ltd. PVB and tetrabutylammonium tetrafluoroborate ($TBABF_4$, purity 98%) were purchased from Vetec and Acros, respectively. Nickel acetate tetrahydrate (purity 98+%), ethanolamine (purity 99%) and high-quality perovskite precursors including lead bromide ($PbBr_2$, purity 99.99%), lead iodide ($PbI_2$, purity 99.9985%) and cesium iodide (CsI, purity 99.9%) were all bought from Alfa Aesar. Formamidinium iodide (FAI, purity 98%) and methylammonium bromide (MABr, purity 98%) were acquired from STAREK Scientific Co. Ltd. and TCI, respectively. [6,6]-Phenyl-$C_{61}$-butyric acid methyl ester ($PC_{61}BM$, purity 99%) and polyethyleneimine (PEI, molecular weight 25,000 g/mol) were obtained from Solenne B. V. and Sigma-Aldrich, respectively. Other solvents were acquired from Alfa Aesar or Acros and utilized without additional purification.

*2. Preparation of perovskite layers*

The composition of the perovskite in this research is $Cs_{0.05}FA_{0.81}MA_{0.14}Pb(Br_{0.15}I_{0.85})_3$. To prepare the precursor solution, a mixture of MABr (21.8 mg), $PbBr_2$ (77.1 mg), FAI (190.2 mg), $PbI_2$ (548.6 mg), and CsI (17.5 mg) in 1 mL of a mixed solvent composed of *N*,*N*-dimethylformamide (DMF) and dimethyl sulfoxide (DMSO) with 4:1 volume ratio was heated at 70 °C with stirring in a sealed glass vial for 1 hour, followed by filtration with 0.22 µm PTFE filters. The perovskite precursor solution was spin coated into thin films in a nitrogen-filled glovebox in two steps: the first step was set at 1200 rpm for 10 s and the second step was set at 4500 rpm for 20 s. During the second step, 300 µL of ethyl acetate was dripped on the spinning substrate. The deposited thin film was then annealed at 105 °C for 1 hour to obtain a dark brown perovskite layer.

*3. Device fabrication*

The device structure of PSCs in this study is FTO/o-$NiO_x$, p-$NiO_x$, or p-$NiO_x$/o-$NiO_x$/perovskite/$PC_{61}BM$+$TBABF_4$/PEI/Ag. FTO-coated glass substrates were cleaned in advance in detergent, deionized water, acetone, and isopropanol (IPA) for 10 min each under ultra-sonication, followed by UV-ozone exposure for 25 min. After depositing $NiO_x$ and perovskite layers, the $PC_{61}BM$ solution (20 mg $mL^{-1}$) containing 2 mol% of $TBABF_4$ in chlorobenzene was spin-coated on the perovskite layer at 3000 rpm for 30 s, followed by heating at 100 °C for 10 min. 0.1 mol% of PEI in anhydrous IPA was then spin-coated on the PCBM layer at 5000 rpm for 30 s. Finally, 100 nm of Ag electrodes were deposited by thermal evaporation at a base pressure of $6 \times 10^{-6}$ torr. The active area of each device was defined by a shadow mask with an open

area of 4.5 mm$^2$. Besides, hole-only devices with the configuration of FTO/o-$NiO_x$, p-$NiO_x$, or p-$NiO_x$/o-$NiO_x$/Ag were fabricated and their current–voltage (*I*–*V*) characteristics were measured for comparison.

*4. Characterization*

*Characterization of o-$NiO_x$ and p-$NiO_x$ films.* The current−voltage (I−V) characteristics of hole-only devices were tested by using a Keithley 2400 source meter. The absorption and transmission spectra of specimens were measured by a Princeton Instruments Acton 2150 spectrophotometer, using a Xenon lamp as the light source (ABET Technologies LS 150). X-ray photoelectron spectroscopy (XPS) measurements were implemented by a Thermo Scientific K-Alpha spectrometer for elemental composition determination of o-NiOxand p-$NiO_x$ films. Ultraviolet photoelectron spectroscopy (UPS) measurements for different $NiO_x$ HTLs were executed on a Thermo VG-Scientific/Sigma Probe spectrometer. He I (hν = 21.22 eV) discharge lamp was used as the excitation source. The top-view morphologies of samples were observed with an ultrahigh resolution ZEISS AURIGA Crossbeam scanning election microscope (SEM). The surface morphology and roughness of different $NiO_x$ films were measured by a Bruker Innova atomic force microscope (AFM). The X-ray diffraction (XRD) patterns and crystallinity were characterized by a Rigaku D/MAX2500 X-ray diffractometer.

*Investigation of the Perovskite layer.* Top-view SEM images were observed with an ultrahigh-resolution ZEISS AURIGA Crossbeam SEM. The steady-state photoluminescence (PL) were acquired by using an Oxford Instruments ANDOR Kymera 193i-B1 spectrometer. An Omicron QuixX 473-100 PS laser with an excitation wavelength at 473 nm was utilized as the light source. To perform time-

resolved PL (TR-PL) measurements, a 473 nm pulsed laser (Omicron) was utilized as an excitation light source. The TR-PL signals were recorded by a time-correlated single-photon counting module (PicoQuant MultiHarp 150 4N) combined with a photomultiplier tube through an Andor Kymera 328i spectrometer.

*Evaluation of solar devices.* The current density−voltage (J−V) characteristics of PSCs were gauged using a Keithley 2400 source meter under AM 1.5G simulated sunlight exposure (Yamashita Denso YSS-100A equipped with a Xenon Short Arc lamp 1000W) at 100 $mW/cm^2$ in ambient environment. The dark current measurement of PSCs was performed using the same source meter in the dark environment. The external quantum efficiency (EQE) spectra were measured by a PV Measurement QE-R instrument.

Table S1. Lifetime parameters of TR-PL curves of the perovskite on the FTO substrate, o-$NiO_x$, p-$NiO_x$, and p-$NiO_x$/o-$NiO_x$ structure.

| substrate | $A_1$ (%) | $\tau_1$ (ns) | $A_2$ (%) | $\tau_2$ (ns) | $\tau_{avg}$ (ns) |
|---|---|---|---|---|---|
| **FTO** | 41.2 | 16.60 | 58.7 | 96.64 | 84.14 |
| **o-$NiO_x$** | 57.5 | 11.12 | 42.4 | 54.68 | 45.25 |
| **p-$NiO_x$** | 57.7 | 9.50 | 42.3 | 62.81 | 53.73 |
| **p-$NiO_x$/o-$NiO_x$** | 50.8 | 9.04 | 49.1 | 36.76 | 31.15 |

Table S2. Device performance of inverted PSCs based on the o-$NiO_x$, p-$NiO_x$ and p-$NiO_x$/o-$NiO_x$ films as the HTL in the reverse and forward scans.

| HTL | Scan | $J_{SC}$ (mA/cm$^2$) | $V_{OC}$ (V) | FF (%) | PCE (%) | HI |
|---|---|---|---|---|---|---|
| o-$NiO_x$ | Reverse | 22.7 | 0.90 | 72 | 14.8 | 0.11 |
| | Forward | 22.1 | 0.99 | 65 | 13.0 | |
| p-$NiO_x$ | Reverse | 21.0 | 1.01 | 66 | 14.2 | 0.15 |
| | Forward | 21.0 | 0.94 | 59 | 12.1 | |
| p-$NiO_x$/o-$NiO_x$ | Reverse | 21.5 | 1.01 | 75 | 15.6 | 0.09 |
| | Forward | 22.3 | 1.00 | 62 | 14.2 | |

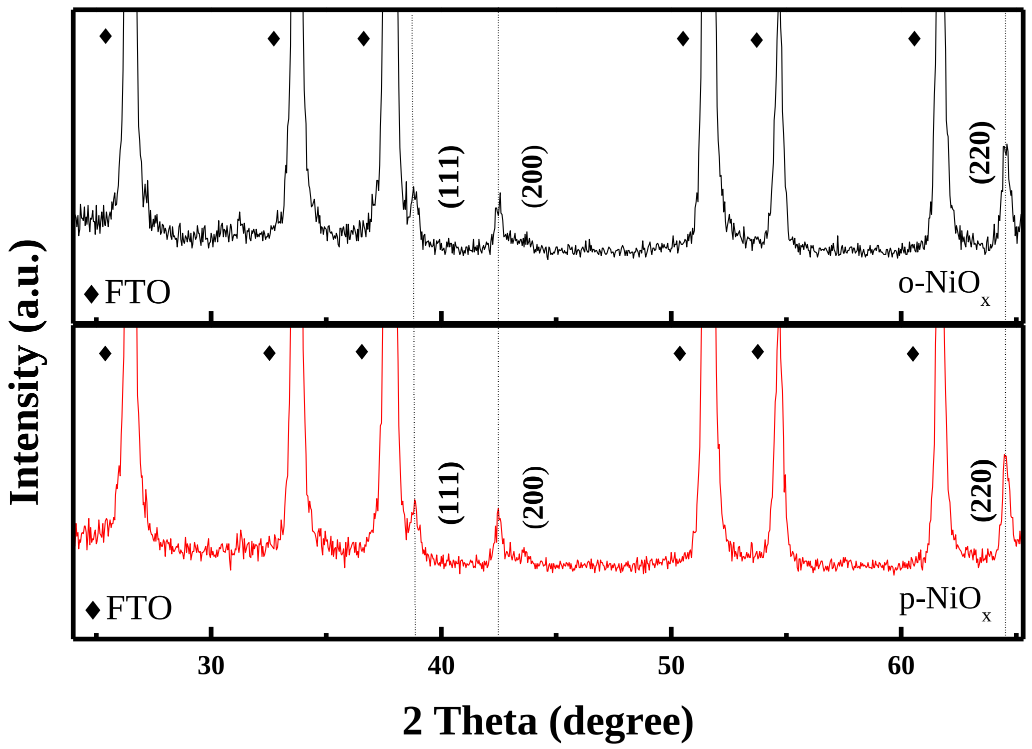


Figure S1. XRD patterns of the o-$NiO_x$ and p-$NiO_x$ films on the FTO substrates.

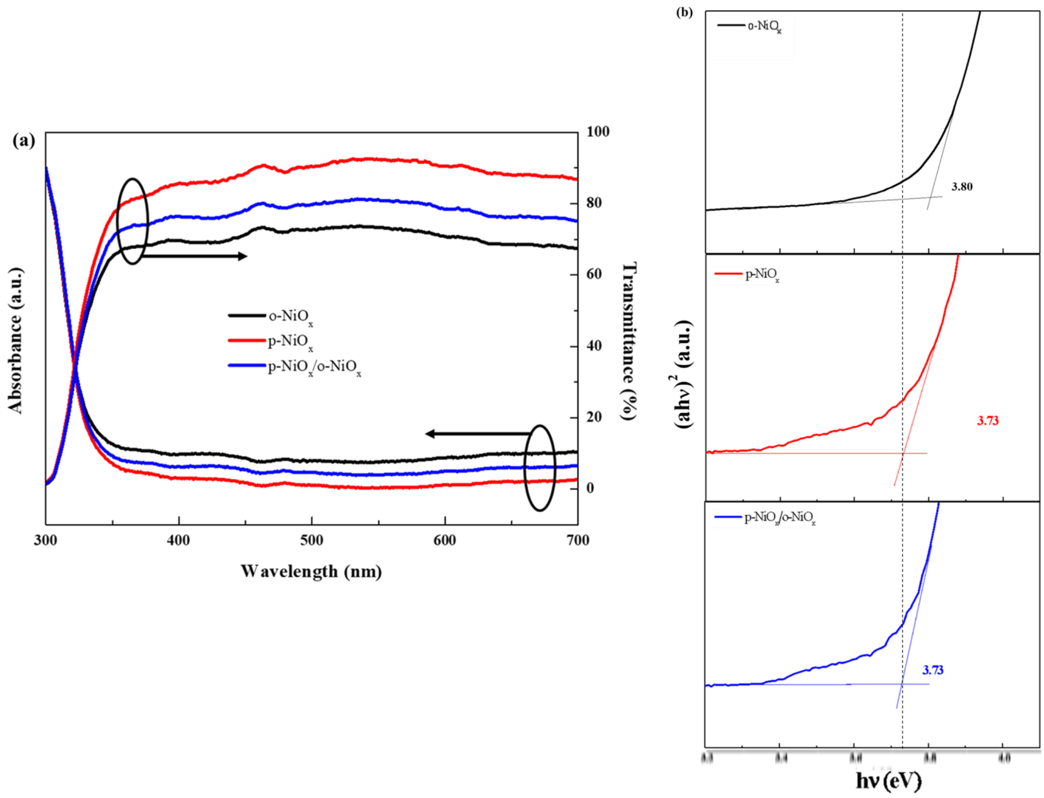


Figure S2. (a) Absorption and transmission spectra, (b) Tauc plots of the o-$NiO_x$, p-$NiO_x$ and p-$NiO_x$/o-$NiO_x$ films deposited on the FTO substrates.

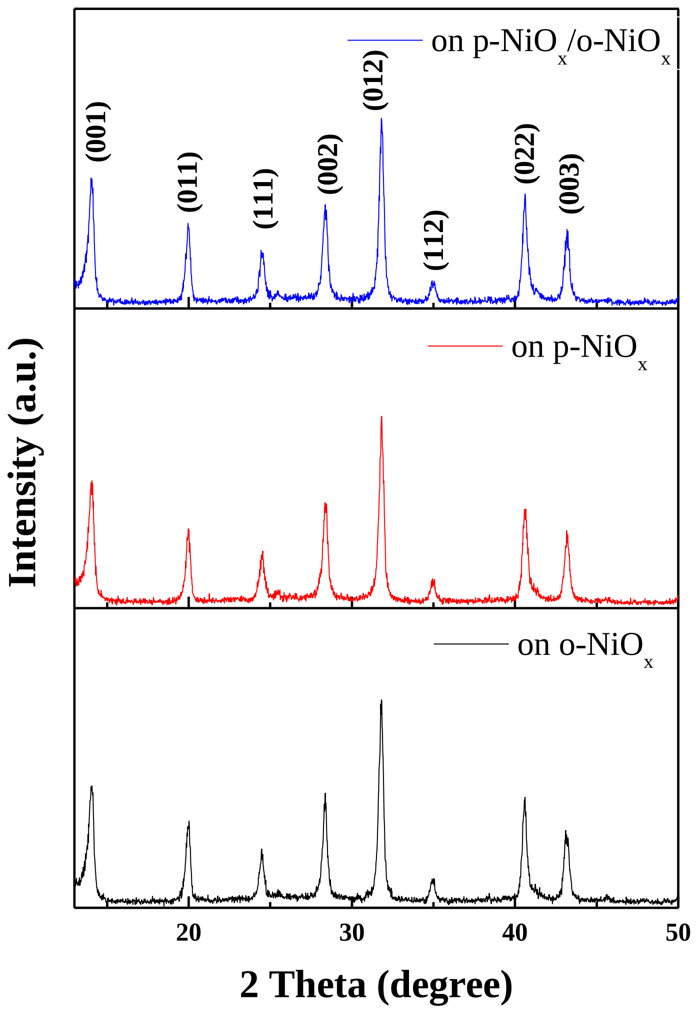


Figure S3. XRD patterns of perovskite layers on the o-$NiO_x$, p-$NiO_x$ and p-$NiO_x$/o-$NiO_x$ films.

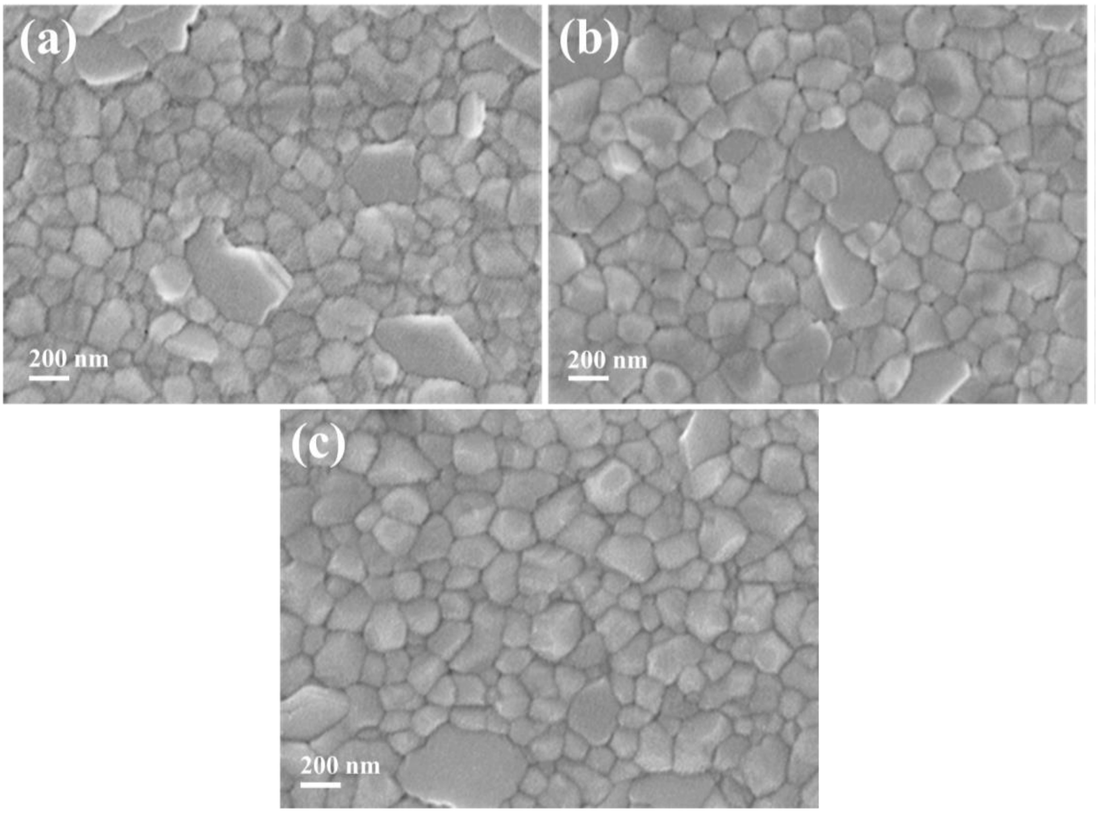


Figure S4. Top-view SEM images of perovskite layers on the (a) o-$NiO_x$, (b) p-$NiO_x$, and (c) p-$NiO_x$/o-$NiO_x$ films.

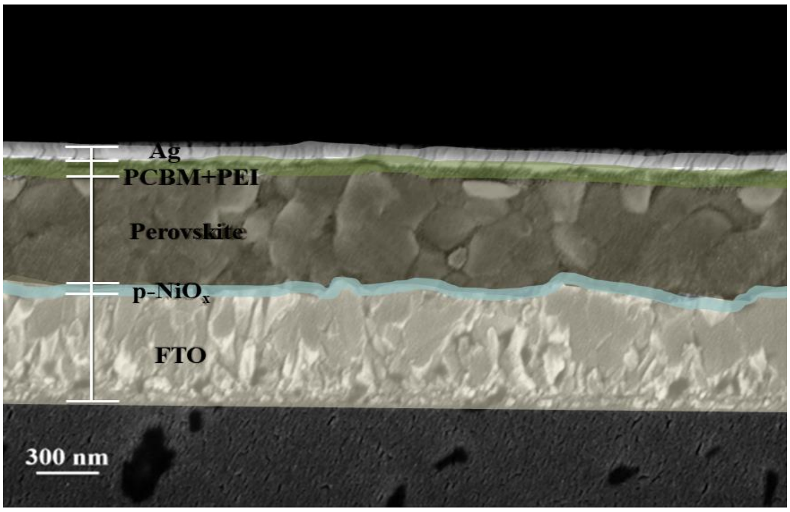


Figure S5. Cross-sectional SEM micrograph of the whole device with p-$NiO_x$.

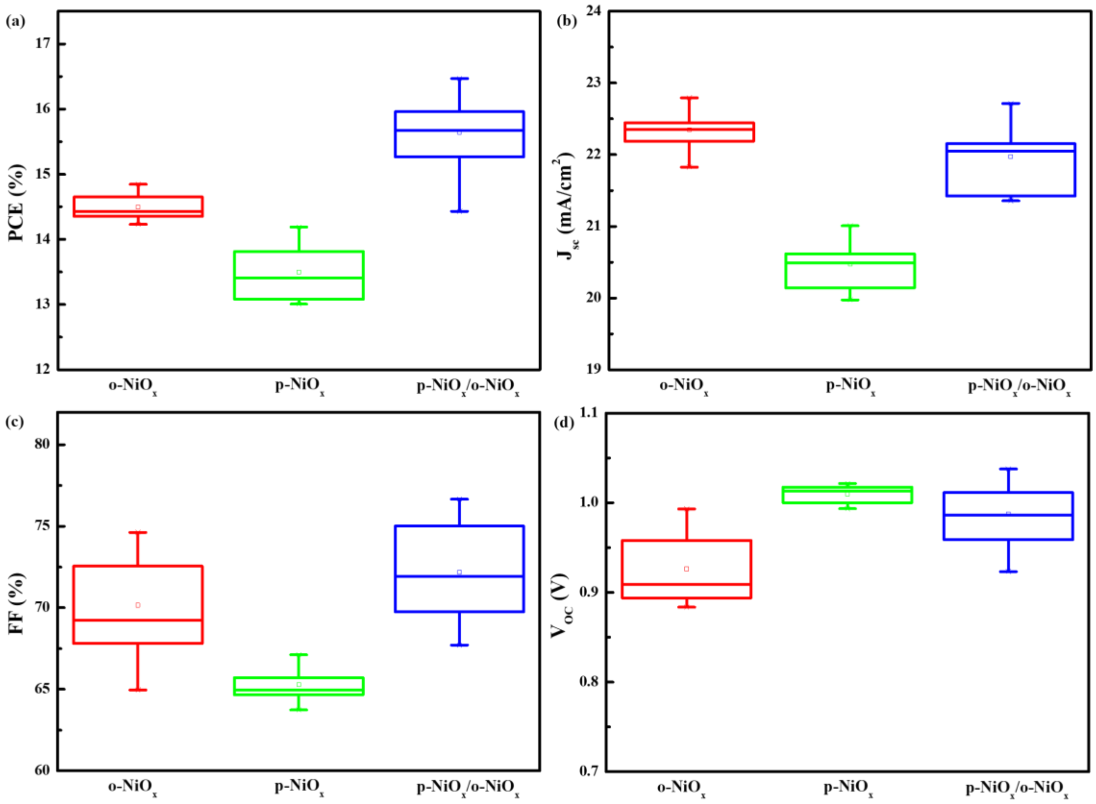


Figure S6. Performance variation is represented as a standard box plot in (a) PCE, (b) $J_{SC}$, (c) FF, and (d) $V_{OC}$ from 20 devices based on the o-$NiO_x$, p-$NiO_x$ and p-$NiO_x$/o-$NiO_x$ films.

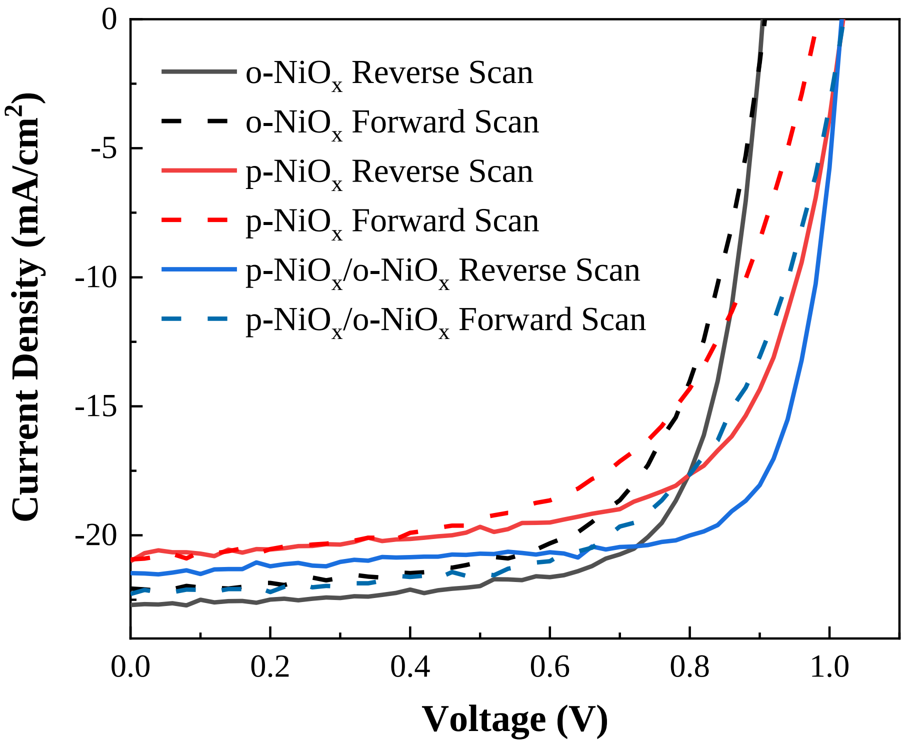


Figure S7. J−V characteristics of PSCs based on the o-$NiO_x$, p-$NiO_x$ and p-$NiO_x$/o-$NiO_x$ HTLs in the reverse and forward scans under AM 1.5G exposure.

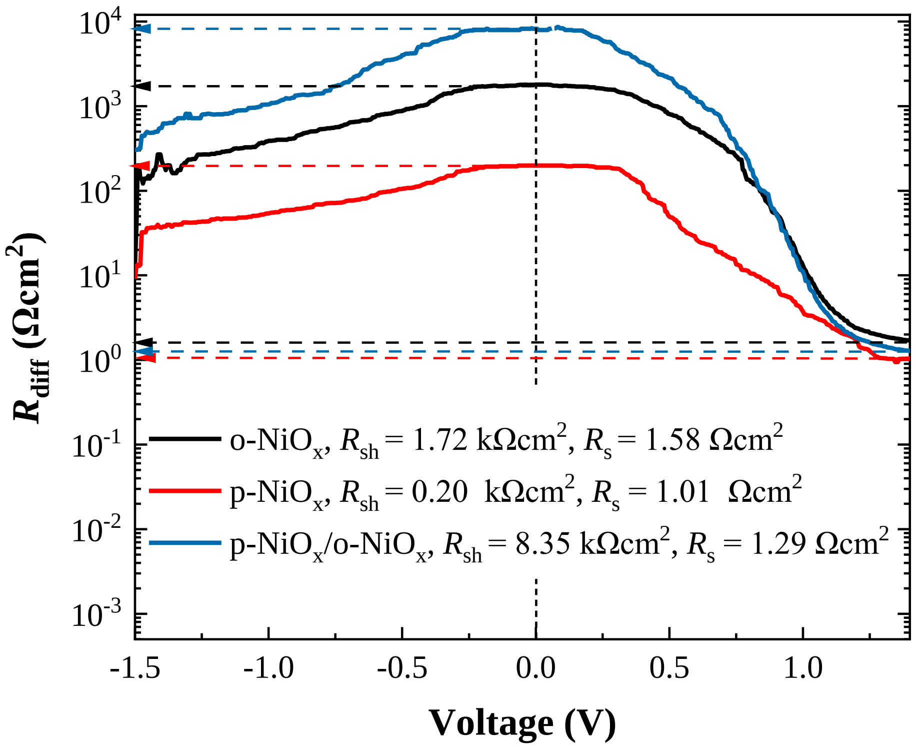


Figure S8. Differential resistance $R_{\mathrm{diff}}$ versus voltage of devices in the dark.
